# CT-Based Quantitative Analysis of Ossification Centres in the C7 Vertebra of Human Fetuses

**DOI:** 10.3390/brainsci15091018

**Published:** 2025-09-20

**Authors:** Magdalena Grzonkowska, Michał Kułakowski, Karol Elster, Bartłomiej Hankiewicz, Michał Janiak, Agnieszka Rogalska, Milena Świtońska, Andrzej Żytkowski, Mariusz Baumgart

**Affiliations:** 1 Department of Normal Anatomy, The Ludwik Rydygier Collegium Medicum in Bydgoszcz, The Nicolaus Copernicus University in Torun, 87-100 Torun, Poland; 2 Clinical Department of Orthopedics and Traumatology, Jan Biziel University hospital nr 2 in Bydgoszcz, Nicolaus Copernicus University in Torun, 87-100 Torun, Poland; mkulakowski@poczta.fm (M.K.); karol.elster@gmail.com (K.E.); michal.janiak@biziel.pl (M.J.); agnieszka.rogalska@biziel.pl (A.R.); 3 Department of Neurology and Clinical Neurophysiology, Jan Biziel University Hospital No. 2, Collegium Medicum in Bydgoszcz, Nicolaus Copernicus University in Torun, Ujejskiego Street 75, 85-168 Bydgoszcz, Poland; m.switonska@cm.umk.pl; 4 Department of Anatomy, Faculty of Medicine, University of Social Sciences in Lodz, 90-113 Lodz, Poland; andrzej.zytkowski.anat@gmail.com; 5 Norbert Barlicki Memorial Teaching Hospital No. 1 of the Medical University of Lodz, 90-113 Lodz, Poland; 6 Faculty of Health Sciences, Powiślański University in Kwidzyn, 82-500 Kwidzyn, Poland

**Keywords:** fetus, ossification center, vertebral body, cervical vertebra, neural process

## Abstract

**Objectives**: The present study aimed to analyze the growth dynamics of the ossification centers of the seventh cervical (C7) vertebra in the human fetus, focusing on linear, planar, and volumetric parameters of both the vertebral body and neural processes. **Methods**: The study was conducted on 55 human fetuses of both sexes (27 males and 28 females), aged 17–30 weeks’ gestation. High-resolution computed tomography, three-dimensional reconstruction, digital image analysis, and appropriate statistical modeling were used to obtain detailed morphometric measurements of the C7 ossification centers. **Results**: All morphometric parameters—length, cross-sectional area, and volume—of the vertebral body ossification center increased linearly with gestational age, except for the sagittal diameter, which followed a logarithmic growth pattern. Linear growth was likewise observed in all diameters of the neural process ossification centers, including length, width, cross-sectional area, and volume. No statistically significant sex-related or side-related differences were detected. **Conclusions**: The CT-based morphometric data and growth models for the ossification centers of C7 presented in this study offer preliminary reference values for the vertebra prominens during fetal development. Although limited by sample size, these results establish a baseline that may assist anatomists, radiologists, obstetricians, pediatricians, and spinal surgeons in assessing cervical-spine maturation and in detecting congenital anomalies prenatally. Further studies involving larger and more diverse fetal cohorts are warranted to validate and extend these observations.

## 1. Introduction

Prenatal development of the vertebral column constitutes a pivotal element of human embryogenesis, underpinning both the stability of the axial skeleton and the proper functioning of the nervous system. This process begins around the third embryonic week with the formation of somites, which subsequently differentiate into sclerotomes—primordial structures that give rise to the vertebrae and ribs [1]. In later stages, vertebral ossification proceeds through two principal mechanisms: endochondral (intracartilaginous) ossification and intramembranous ossification, with the former accounting for the development of most vertebrae [2].

Each vertebra originates from three primary ossification centers: one located in the vertebral body and two in the neural processes. Ossification of the neural processes begins around the eighth gestational week in the upper cervical vertebrae and progresses caudally, whereas vertebral body ossification begins around the tenth week, initially in the lower thoracic and upper lumbar regions, and spreads in both cranial and caudal directions [3].

Accurate knowledge of the chronology and dynamics of vertebral ossification is of paramount importance across numerous medical disciplines. In prenatal diagnostics, it enables early detection of congenital anomalies such as spina bifida, sacral agenesis, and skeletal dysplasias [4]. In orthopedics and neurology, it facilitates assessment of fetal skeletal maturity and identification of potential musculoskeletal and neurodevelopmental disorders. Moreover, in prenatal radiology, detailed ossification data are indispensable for interpreting images obtained through ultrasonography, computed tomography, and magnetic resonance imaging [5].

C7, known as the vertebra prominens, constitutes the transitional point between the mobile cervical spine and the more rigid thoracic spine. Its long, non-bifid spinous process makes it easily palpable and frequently used as a surface landmark, although studies indicate that palpation alone often localizes C7 inaccurately, necessitating imaging guidance [6,7]. Unique morphological characteristics—such as robust pedicles and laminae, and typically narrow transverse foramina that do not transmit the vertebral artery—grant C7 a central role in cervical spine biomechanics and surgical stabilization procedures [8,9].

Morphometric variability in this region has been well documented, particularly between the C5 and C7 levels, where in adult populations it has been associated with degenerative changes [10]. Postnatal studies consistently report that vertebral dimensions increase progressively from C3 to C7, with C7 showing the largest transverse and laminar measurements [8,11]. In contrast, in our fetal series, the observed variability reflects differences in morphological arrangement. Previous investigations have primarily focused on other vertebrae—for example, Baumgart et al. [12] examined C4 ossification and demonstrated logarithmic growth in selected parameters. To date, however, no detailed prenatal morphometric analysis has specifically addressed the vertebra prominens.

This lack of prenatal data is clinically relevant. Reference values for C7 during fetal development would support improved imaging-based diagnosis of congenital spinal anomalies and inform surgical planning. Given that 7–14% of pediatric cervical fractures occur at the C7–T1 level, accurate identification and evaluation of this region is essential for trauma diagnostics [13].

Against this background, the present study analyzes the ossification of the vertebral body and neural processes of the seventh cervical vertebra in human fetuses using computed tomography. The aim of the study was to investigate potential sex-related and side-related differences in all analyzed parameters, to conduct a quantitative assessment of the ossification centers of C7—including linear, planar, and volumetric measurements—to establish preliminary normative values for successive gestational weeks, and to characterize the growth dynamics of all parameters by developing mathematical models with the best possible fit to the observed data.

## 2. Materials and Methods

### 2.1. Examined Sample

The study material comprised 55 human fetuses—27 males and 28 females—ranging from 17 to 30 weeks of gestation. The specimens, obtained from spontaneous miscarriages and preterm deliveries before 2000, are curated in the Department of Normal Anatomy, Collegium Medicum in Bydgoszcz, Nicolaus Copernicus University in Toruń. Ethical approval was granted by the Bioethics Committee of Collegium Medicum (KB 275/2011). All procedures conformed to Polish legislation under the Body Donation Program for adults and fetuses and adhered to the principles of the Declaration of Helsinki.

Fetuses displaying well-preserved external morphology and documented clinical histories were included. Specimens with overt malformations, musculoskeletal developmental disorders, intrauterine growth restriction, or other major congenital anomalies were excluded.

Gestational age was determined based on both crown–rump length (CRL) and the date of the mother’s last menstrual period (LMP). Only fetuses demonstrating high concordance between CRL-based and LMP-based estimates (Pearson’s r = 0.98, *p* < 0.001) were included in the analysis. Table 1 summarizes the sample characteristics by gestational week and sex.

### 2.2. Morphometric Measurements and Assessment of Ossification Centers

**Figure 1:**
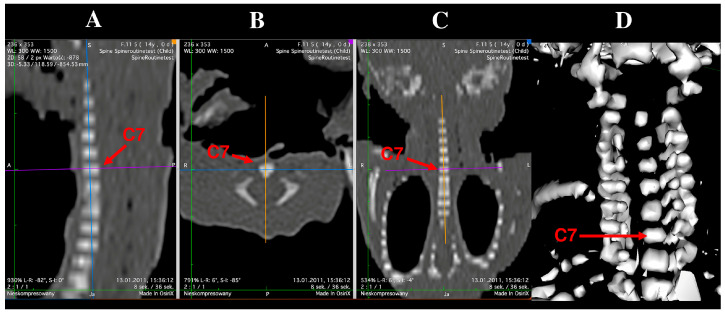
Multiplanar reconstructions (MPR) of the C7 vertebra in sagittal (**A**), transverse (**B**), and frontal (**C**) projections, and volumetric 3D reconstruction (**D**).

Computed tomography (CT) scans were acquired using a Siemens Biograph 128 mCT scanner (Siemens Healthcare GmbH, Erlangen, Germany) at the Department of Positron Emission Tomography and Molecular Imaging, Oncology Center, Collegium Medicum, Bydgoszcz, Poland. Images were stored in DICOM format with an interslice spacing of 0.4 mm (Figure 1).

CT scanning was performed using the following acquisition parameters: 60 mAs, 80 kV, a pitch of 0.35, a field of view of 180 mm, and a rotation time of 0.5 s. Image reconstruction was carried out with a slice thickness of 0.4 mm, a reconstruction increment of 0.6 mm, and a B45 f-medium convolution kernel. The resulting grayscale values (Hounsfield units, HU) ranged from −275 to −134 (minimum) and from +1165 to +1558 (maximum), yielding a window width of 1404–1692 HU and a window level ranging from +463 to +712 HU.

**Figure 2:**
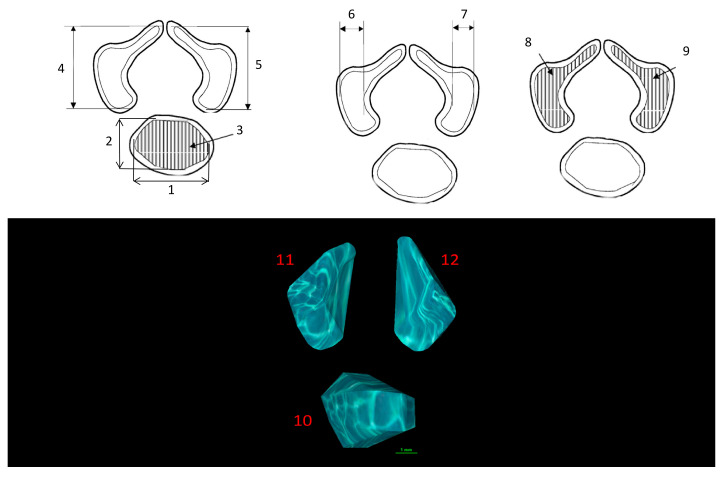
Diagram illustrating the measurement protocol for the ossification centers of the C7 vertebra.

Ossification centers of the vertebral body and neural processes of the seventh cervical (C7) vertebra were measured according to a predefined protocol (Figure 2). For each fetus, linear diameters, cross-sectional areas, and volumes were recorded. Despite the cartilaginous stage, the clearly defined bony contours allowed for reliable sagittal, transverse, and volumetric assessment [14,15]. The CT datasets were used to create three-dimensional reconstructions of the C7 ossification centers, which were subsequently subjected to morphometric analysis using Osirix 3.9 MD software. It should be emphasized that Osirix 3.9 MD enables precise numerical evaluation of linear, surface, and volumetric parameters based on high-resolution multiplanar and 3D reconstructions of the studied anatomical structures.

Measured variables:

1. Transverse diameter of the vertebral body ossification center—distance between the lateral margins in the transverse plane (Figure 2);

2. Sagittal diameter of the vertebral body ossification center—distance between the anterior and posterior margins in the transverse plane (Figure 2);

3. Cross-sectional area of the vertebral body ossification center—calculated by tracing the outline in the transverse plane (Figure 2);

4–5. Length of the right and left neural process ossification centers—distance between the proximal and distal margins in the transverse plane (Figure 2);

6–7. Width of the right and left neural process ossification centers (Figure 2);

8–9. Cross-sectional area of the right and left neural process ossification centers—calculated by tracing the outline in the transverse plane (Figure 2);

10–12. Volume of each ossification center—computed using advanced imaging software that enables three-dimensional reconstruction and accounts for spatial orientation and X-ray attenuation of osseous tissue (Figure 2).

### 2.3. Statistical Analysis

Data were analyzed using Statistica 12.5 and PQStat 1.6.2. Normality was assessed with the Shapiro–Wilk test, and homogeneity of variance was verified using Fisher’s test. Side-to-side differences were evaluated with the paired Student’s t-test, while sex-related differences were assessed using the unpaired Student’s t-test. One-way ANOVA followed by Tukey’s post hoc test was applied; when variances were unequal, the Kruskal–Wallis test was used. Growth dynamics were modeled using linear and nonlinear regression, with the goodness of fit evaluated by the coefficient of determination (R^2^). Statistical significance was set at *p* < 0.05. Relationships among variables were quantified using Pearson’s correlation coefficient (r).

Each measurement was performed three times under identical conditions (M.B.), and the mean value was used for analysis. Each measurement was taken three times under the same conditions but at different times (with one-day intervals) and then averaged. As shown in Table 2, intraclass correlation coefficients (ICC) calculated for a single observer (M.B.) were highly significant (*p* < 0.001), demonstrating excellent repeatability.

## 3. Results

Mean values and standard deviations of the parameters investigated for the ossification centers of the C7 vertebral body and neural processes in human fetuses during the analyzed developmental period are presented in Table 3, Table 4 and Table 5. Statistical analysis revealed no significant sex-related or side-related differences, thereby justifying the construction of a single growth curve for each parameter examined.

### 3.1. Morphometric Parameters of the Ossification Center of the C7 Vertebral Body

The developmental dynamics of the transverse diameter, cross-sectional area, and volume of the C7 vertebral body ossification center followed a linear function—that is, they were directly proportional to fetal age—whereas the sagittal diameter followed a logarithmic function.

**Figure 3:**
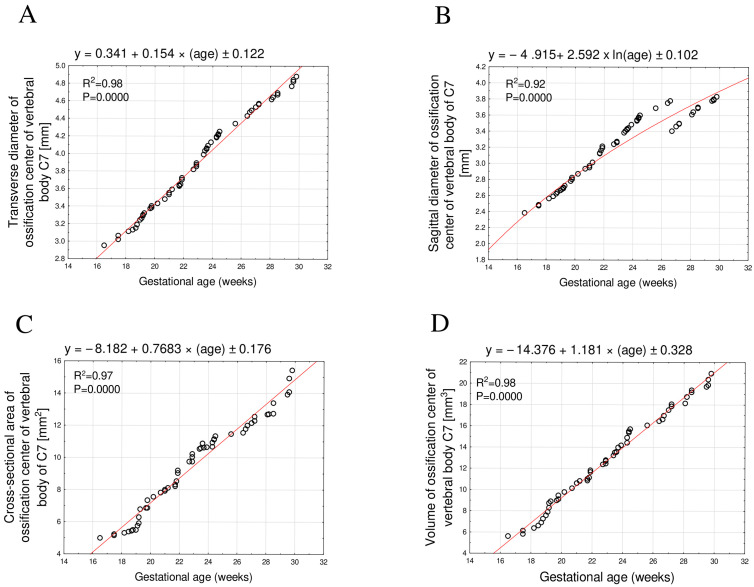
Regression lines for transverse diameter (**A**), sagittal diameter (**B**), cross-sectional area (**C**), and volume (**D**) of the ossification center of the C7 vertebral body.

The mean transverse diameter of the C7 vertebral body ossification center between 17 and 30 gestational weeks ranged from 2.95 mm to 4.83 ± 0.05 mm, showing strictly proportional growth described by the equation y = 0.341 + 0.154 × age ± 0.122, R^2^ = 0.98 (Figure 3A).

The mean sagittal diameter over the same period ranged from 2.38 mm to 3.80 ± 0.02 mm and was best described by a logarithmic model: y = −4.915 + 2.592 × ln(age) ± 0.102, R^2^ = 0.92 (Figure 3B).

The mean cross-sectional area increased from 4.99 mm^2^ to 14.58 ± 0.71 mm^2^, fitting a linear model: y = −8.182 + 0.768 × age ± 0.176, R^2^ = 0.97 (Figure 3C).

The mean volume rose from 5.60 mm^3^ to 20.20 ± 0.57 mm^3^, likewise following a directly proportional increase: y = −14.376 + 1.181 × age ± 0.328, R^2^ = 0.98 (Figure 3D).

### 3.2. Morphometric Parameters of the Right and Left C7 Neural Process Ossification Centers

The developmental dynamics of the length, width, cross-sectional area, and volume of the right and left C7 neural process ossification centers also followed a linear pattern and were therefore directly proportional to fetal age.

**Figure 4:**
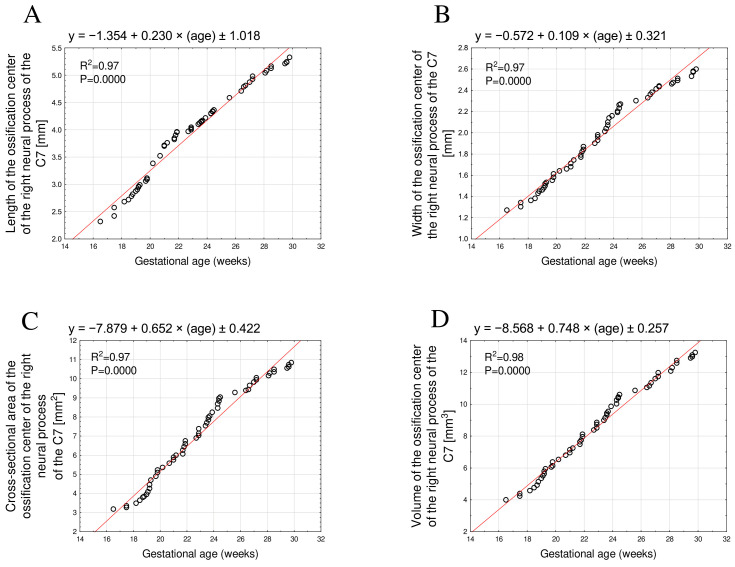
Regression lines for length (**A**), width (**B**), cross-sectional area (**C**), and volume (**D**) of the ossification center of the right C7 neural process.

**Figure 5:**
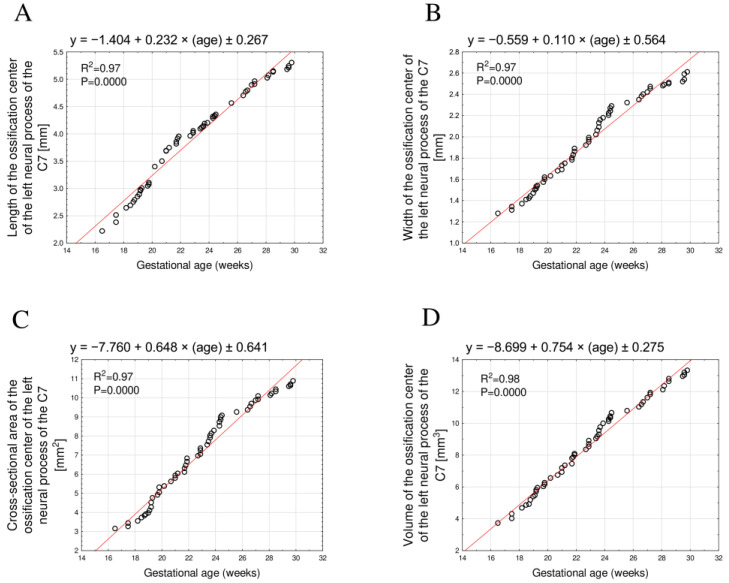
Regression lines for length (**A**), width (**B**), cross-sectional area (**C**), and volume (**D**) of the ossification center of the left C7 neural process.

The mean length of the neural process ossification center increased from 2.32 mm to 5.25 ± 0.05 mm on the right and from 2.22 mm to 5.23 ± 0.05 mm on the left, fitting the linear models y = −1.354 + 0.230 × age ± 1.018, R^2^ = 0.97 (Figure 4A) and y = −1.404 + 0.232 × age ± 0.267, R^2^ = 0.97 (Figure 5A), respectively.

The mean width varied from 1.27 mm to 2.57 ± 0.03 mm on the right side and from 1.28 mm to 2.57 ± 0.04 mm on the left side, following the equations y = −0.572 + 0.109 × age ± 0.321, R^2^ = 0.97 (Figure 4B), and y = −0.559 + 0.110 × age ± 0.564, R^2^ = 0.97 (Figure 5B), respectively.

The mean cross-sectional area increased from 3.17 mm^2^ to 10.69 ± 0.13 mm^2^ on the right and from 3.15 mm^2^ to 10.70 ± 0.12 mm^2^ on the left, in accordance with the linear models y = −7.879 + 0.652 × age ± 0.422, R^2^ = 0.97 (Figure 4C), and y = −7.760 + 0.648 × age ± 0.641, R^2^ = 0.97 (Figure 5C), respectively.

Finally, the mean volume increased from 3.99 mm^3^ to 13.04 ± 0.15 mm^3^ on the right and from 3.73 mm^3^ to 12.70 ± 0.11 mm^3^ on the left, conforming to the linear relationships y = −8.568 + 0.748 × age ± 0.257, R^2^ = 0.98 (Figure 4D), and y = −8.699 + 0.754 × age ± 0.275, R^2^ = 0.98 (Figure 5D), respectively.

## 4. Discussion

To the best of our knowledge, this is the first study to provide a comprehensive, CT-based quantitative assessment of all ossification centers of the C7 in human fetuses, encompassing both the vertebral body and neural processes with linear, planar, and volumetric measurements, and modeling gestational age-specific growth trajectories. The results obtained for C7, when compared with previously published data on the cervical spine, clearly demonstrate a gradual shift in growth kinetics along the cranio-caudal axis. The most cranial vertebrae—the atlas and axis—undergo the most rapid mineralisation: the length and width of their neural processes increase logarithmically, whereas their cross-sectional areas and volumes enlarge linearly [16,17]. In the fourth cervical vertebra, growth kinetics are mixed: the principal diameters of the vertebral body increase logarithmically, whereas cross-sectional areas and volume follow linear or fourth-degree polynomial trajectories [12]. The cross-sectional analysis by Szpinda et al. [18,19], which encompassed vertebral bodies and neural arches from C1 to S5, corroborates a generally linear enlargement of areas and volumes but simultaneously documents a progressive decline in absolute values toward the lumbar region, reflecting functional differentiation of individual segments.

Against this background, C7 exhibits a distinctive, predominantly linear growth pattern across all analyzed parameters, with the sole exception of the sagittal diameter of the body, which—analogous to C4 [12]—shows a nonlinear profile. This pattern suggests that the cervicothoracic transitional vertebra maintains a proportional increase in size throughout the examined span of the second trimester, presumably to meet the escalating mechanical demands imposed on the cervicothoracic junction.

Neither sex-related nor side-related variability emerged in our C7 dataset, a finding that concurs with all comparable fetal CT studies conducted to date [12,16,17,18,19]. Baumgart et al. [12] detected no significant sexual dimorphism or lateral asymmetry in the ossification centres of C4 and, later, of the atlas and axis [16,17], while Szpinda et al. [18,19] likewise reported bilateral and inter-sex homogeneity for neural and vertebral-body centres throughout the C1–S5 column. Taken together with our results, this body of evidence indicates that, during the second trimester, mineralisation of cervical vertebrae proceeds in a remarkably symmetrical and sex-independent manner. From a practical standpoint, the absence of early dimorphism supports the use of a single, unified set of reference ranges for routine prenatal assessment of ossification timing and size. It also suggests that the sex-specific growth disparities documented in post-natal cohorts arise only after birth—most likely under the influence of hormonal surges and biomechanical loading—rather than representing an intrinsic feature of fetal skeletogenesis.

Post-natal cadaveric series consistently show a stepwise rise in transverse diameter and laminar thickness from C3 to C7, with peak values at C7 [8,11]. The current fetal results mirror that trend, indicating that the morphometric distinctiveness of the vertebra prominens is established well before birth and is not merely the consequence of post-natal mechanical loading. Accordingly, our nomograms supply a developmental baseline against which later degenerative or dysplastic remodelling of C7 can be evaluated.

Beyond anatomy, the study reinforces the diagnostic merit of fetal CT. Victoria et al. [20] demonstrated that low-dose CT uncovers clinically significant skeletal abnormalities overlooked by ultrasonography in 81% of severe dysplasia cases and yields a four-to-five-fold lower measurement error (≈ 1.8% vs. ≈ 8.7%). Our findings extend this evidence to the cervicothoracic junction, showing that even cartilaginous C7 centres can be segmented reliably for volumetric analysis. In everyday fetal practice, such normative data may improve age estimation when standard biometric parameters are ambiguous, sharpen the recognition of segmentation defects or mineralisation delay, and guide multidisciplinary counselling.

Clinical relevance extends into the perinatal and early paediatric periods. Accurate prenatal morphometry of C7 can inform surgical planning for congenital cervical kyphosis, hemivertebra excision or posterior instrumentation, where the small calibre of paediatric pedicles poses a technical challenge. Post-traumatic management may also benefit, because 7–14% of paediatric cervical fractures involve the C7–T1 level, and knowledge of expected ossification volumes may aid in differentiating fracture from normal cartilaginous clefts on imaging [13].

Technological progress promises to widen the indication spectrum. Iterative and deep-learning reconstruction algorithms now reduce image noise at radiation doses < 3 mGy, below accepted fetal thresholds, while maintaining sub-millimetre spatial resolution. Moreover, artificial-intelligence pipelines for automatic vertebral labelling and centre-line extraction could ultimately provide real-time nomogram overlays during obstetric scans, enabling immediate identification of outliers and more consistent referral pathways.

### Limitations of the Study

A relevant limitation of this study is the restricted gestational age range of the cases (17–30 weeks). Due to the limited availability of fetal material, we could not analyze each gestational week separately. Consequently, the nomograms should be viewed as preliminary. A multicentre effort covering earlier embryonic stages, the late third trimester and a larger ethnic spectrum would strengthen external validity. Longitudinal correlation with neonatal and infant imaging is likewise warranted to determine how early deviations from our curves translate into post-natal pathology.

## 5. Conclusions

1. No sex-related or laterality differences were observed in any of the morphometric parameters of the vertebral body or primary ossification centers of the C7 vertebra.

2. All examined dimensions of the C7 vertebral body and its ossification centers increased with advancing gestational age between 17 and 30 weeks. Most parameters exhibited a linear growth pattern, while the sagittal diameter of the vertebral body ossification center followed a logarithmic trend.

3. The morphometric values obtained for the C7 vertebra in this study provide preliminary, gestational age-specific reference data that may support fetal age estimation and assist in the ultrasonographic or tomographic evaluation of congenital anomalies affecting the lower cervical spine. While these findings offer a valuable baseline, validation in larger, more diverse cohorts—including both earlier and later gestational stages—is necessary to refine these initial observations and fully determine their clinical utility.

## Figures and Tables

**Table 1:** Age, number and sex of the fetuses studied.

Gestational Age	Crown–Rump Length (mm)				Number of Fetuses	Sex	
Weeks (Hbd-Life)	Mean	SD	Min.	Max.		♂	♀
17	115.00	-	115.00	115.00	1	0	1
18	133.33	5.77	130.00	140.00	3	1	2
19	149.50	3.82	143.00	154.00	8	3	5
20	161.00	2.71	159.00	165.00	4	2	2
21	174.75	2.87	171.00	178.00	4	3	1
22	185.00	1.41	183.00	186.00	4	1	3
23	197.60	2.61	195.00	202.00	5	2	3
24	208.67	3.81	204.00	213.00	9	5	4
25	214.00	-	214.00	214.00	1	0	1
26	229.00	5.66	225.00	233.00	2	1	1
27	237.50	3.33	233.00	241.00	6	6	0
28	249.50	0.71	249.00	250.00	2	0	2
29	253.00	0.00	253.00	253.00	2	0	2
30	263.25	1.26	262.00	265.00	4	3	1
Total					55	27	28

**Table 2:** Intra-class correlation coefficients (ICC) values for inter-observer recurrence.

Parameter of the Body Ossification Center of C7 Vertebra	ICC
Transverse diameter	0.998 *
Sagittal diameter	0.995 *
Cross-sectional area	0.996 *
Volume	0.997 *
Parameter of the Right Ossification Center of the Neural Process of C7	
Length	0.996 *
Width	0.997 *
Cross-sectional area	0.994 *
Volume	0.998 *
Parameter of the Left Ossification Center of the Neural Process of C7	
Length	0.997 *
Width	0.996 *
Cross-sectional area	0.997 *
Volume	0.997 *

Inter-class correlation coefficients marked with * are statistically significant at *p*  <  0.0001.

**Table 3:** Morphometric parameters of the ossification center of the C7 vertebral body.

GA (Weeks)	N	Ossification Center of the Vertebral Body C7							
		Transverse Diameter (mm)		Sagittal Diameter (mm)		Cross-Sectional Area (mm^2^)		Volume (mm^3^)	
		Mean	SD	Mean	SD	Mean	SD	Mean	SD
17	1	2.95		2.38		4.99		5.60	
18	3	3.06	0.05	2.50	0.05	5.22	0.07	6.10	0.27
19	8	3.24	0.07	2.66	0.04	5.81	0.49	7.76	0.83
20	4	3.40	0.03	2.82	0.04	7.15	0.35	9.31	0.36
21	4	3.54	0.05	2.97	0.03	7.95	0.13	10.53	0.29
22	5	3.67	0.04	3.16	0.04	8.65	0.44	11.30	0.41
23	5	3.88	0.06	3.28	0.06	10.05	0.34	12.70	0.33
24	9	4.13	0.07	3.49	0.07	10.82	0.21	14.31	0.77
25	1	4.25		3.60		11.32		15.67	
26	2	4.39	0.06	3.72	0.04	11.50	0.04	16.24	0.30
27	5	4.52	0.04	3.53	0.15	12.14	0.29	17.38	0.61
28	2	4.63	0.01	3.63	0.02	12.68	0.03	18.44	0.43
29	2	4.68	0.01	3.70	0.01	13.05	0.46	19.27	0.16
30	4	4.83	0.05	3.80	0.02	14.58	0.71	20.20	0.57

**Table 4:** Morphometric parameters of the right ossification center of the C7 neural process.

GA (Weeks)	N	Right Ossification Center of the Neural Process of C7							
		Length (mm)		Width (mm)		Cross-Sectional Area (mm^2^)		Volume (mm^3^)	
		Mean	SD	Mean	SD	Mean	SD	Mean	SD
17	1	2.32		1.27		3.17		3.99	
18	3	2.56	0.13	1.33	0.03	3.37	0.11	4.40	0.17
19	8	2.87	0.09	1.47	0.05	4.08	0.36	5.37	0.42
20	4	3.16	0.15	1.60	0.04	5.13	0.20	6.27	0.21
21	4	3.67	0.11	1.70	0.04	5.79	0.19	7.02	0.20
22	5	3.89	0.06	1.82	0.04	6.41	0.27	7.78	0.26
23	5	4.03	0.05	1.96	0.04	7.19	0.26	8.68	0.25
24	9	4.23	0.09	2.15	0.07	8.30	0.46	9.81	0.48
25	1	4.36		2.27		9.03		10.60	
26	2	4.65	0.09	2.32	0.02	9.33	0.09	10.95	0.12
27	5	4.87	0.08	2.40	0.03	9.78	0.24	11.55	0.32
28	2	5.06	0.03	2.47	0.01	10.22	0.09	12.19	0.16
29	2	5.14	0.03	2.50	0.01	10.42	0.09	12.65	0.10
30	4	5.25	0.05	2.57	0.03	10.69	0.13	13.04	0.15

**Table 5:** Morphometric parameters of the left ossification center of the C7 neural process.

GA (Weeks)	N	Left Ossification Center of the Neural Process of C7							
		Length (mm)		Width (mm)		Cross-Sectional Area (mm^2^)		Volume (mm^3^)	
		Mean	SD	Mean	SD	Mean	SD	Mean	SD
17	1	2.22		1.28		3.15		3.73	
18	3	2.51	0.13	1.34	0.03	3.41	0.14	4.33	0.33
19	8	2.86	0.11	1.48	0.05	4.13	0.36	5.41	0.40
20	4	3.15	0.17	1.61	0.03	5.16	0.21	6.25	0.22
21	4	3.65	0.11	1.71	0.03	5.84	0.18	7.05	0.27
22	5	3.89	0.06	1.83	0.04	6.47	0.28	7.84	0.25
23	5	4.03	0.05	1.97	0.04	7.23	0.24	8.70	0.28
24	9	4.22	0.09	2.17	0.07	8.35	0.45	9.88	0.46
25	1	4.35		2.29		9.07		10.64	
26	2	4.63	0.10	2.34	0.02	9.29	0.08	10.90	0.16
27	5	4.86	0.08	2.42	0.04	9.82	0.21	11.57	0.31
28	2	5.05	0.04	2.49	0.01	10.17	0.07	12.22	0.18
29	2	5.14	0.01	2.51	0.01	10.38	0.09	12.70	0.11
30	4	5.23	0.05	2.57	0.04	10.70	0.12	12.70	0.11

## Data Availability

The original contributions presented in this study are included in the article. Further inquiries can be directed to the corresponding authors.
